# The Effect of Different Treatment Regimens and Multiple Risk Factors on Adverse Pregnancy Outcomes among Syphilis-Seropositive Women in Guangzhou: A Retrospective Cohort Study

**DOI:** 10.1155/2020/7626274

**Published:** 2020-05-01

**Authors:** Fang Hu, Shuai-Jun Guo, Jian-Jun Lu, Sui Zhu, Ning-Xuan Hua, Yan-Yan Song, Jing-Jing Liang, Jia Yu, Sui-Fang Lin

**Affiliations:** ^1^Department of Child Health, Guangzhou Women and Children's Medical Center, Guangzhou Medical University, Guangzhou, Guangdong, China; ^2^Centre for Community Child Health, Murdoch Children's Research Institute, Royal Children's Hospital, 3052 Melbourne, Victoria, Australia; ^3^Department of Pediatrics, University of Melbourne, 3010 Melbourne, Victoria, Australia; ^4^The Second School of Clinical Medicine, Southern Medical University, Guangzhou, Guangdong, China; ^5^Department of Medical Affairs, The First Affiliated Hospital of Sun Yat-sen University, Guangzhou, Guangdong, China; ^6^Department of Medical Statistics, School of Medicine, Jinan University, Guangzhou, Guangdong, China; ^7^Department of Woman Health, Guangzhou Women and Children's Medical Center, Guangzhou Medical University, Guangzhou, Guangdong, China

## Abstract

**Background:**

To eliminate mother-to-child transmission of syphilis, the Chinese government recommends a treatment regimen that slightly differs from the World Health Organization- (WHO-) recommended treatment. However, little is known about their difference in efficacy. This study is aimed at comparing the effect of China-recommended and WHO-recommend treatment regimens on adverse pregnancy outcomes (APOs) and at examining associated risk factors of APOs among syphilis-seropositive women.

**Methods:**

Using the syphilis registry data, we retrospectively collected data from 4488 syphilis-infected pregnant women in Guangzhou during 2011-2018. Multivariate analyses were used to investigate the association between treatment regimens and APOs (ectopic pregnancy, spontaneous abortion, stillbirth, preterm birth or low birth weight, newborn smaller than gestational age, congenital syphilis, and infant death) and the association between risk factors and APOs.

**Results:**

Of 3474 participants, 27.3% had at least one APO. Compared to those receiving WHO-recommended treatment, women who received China-recommended treatment were less likely to have APOs (odds ratio (OR) 0.47, 95% confidence interval (CI) 0.38-0.57), whereas those who received no treatment had 1.6 times higher odds of experiencing APOs. One common risk factor across different APOs was high levels of log_2_-transformed toluidine red unheated serum test (TRUST) titers before treatment (OR 1.14, 95% CI 1.10-1.19). China-recommended treatment was effective in reducing APOs for those with TRUST ≥ 1 : 8 (OR 0.21, 95% CI 0.14-0.29) and those with TRUST < 1 : 8 (OR 0.62, 95% CI 0.50-0.77).

**Conclusions:**

Syphilis-seropositive women receiving China-recommended treatment had lower odds of APOs, especially when TRUST titers before treatment were high. Findings can be used to guide health professionals to reduce APOs among syphilis-infected mothers and promote nationwide use of China-recommended treatment.

## 1. Introduction

Maternal syphilis is a significant public health concern, and elimination of congenital syphilis (CS) is one of the Millennium Development Goals [1]. Estimates show that global maternal syphilis prevalence was 0.69% in 2016 [2]. Mother-to-child transmission (MTCT) of syphilis can occur at any stage of the disease and at any gestational age [3]. Pregnant women with syphilis are at higher risk of adverse pregnancy outcomes (APOs) if they receive no treatment or inadequate treatment [3]. These APOs include miscarriage, stillbirth, preterm birth, LBW, neonatal death, and CS [4].

Currently, benzathine penicillin G (BPG) treatment is widely used as the most effective antenatal care intervention for maternal syphilis, particularly in the first two trimesters [4]. But, guidelines for BPG use differ across countries (Table 1) and mainly depended on the country-specific healthcare systems and contexts. In addition, due to the shortage of penicillin or penicillin allergy, some countries use alternatives to penicillin treatment [5]. The World Health Organization (WHO) [1] and the U.S. Centers for Disease Control and Prevention (CDC) [6] recommend one to three doses of 2.4 million International Units (IU) of BPG at least 30 days before delivery depending on the stages of syphilis, with the efficacy of this therapy being well documented. The British guideline suggests using BPG as a first-line drug and ceftriaxone as an alternative for pregnant women who are allergic to penicillin [7]. The Chinese Maternal and Child Health Services Division of the National Health Commission recommends two courses of penicillin in the first and third trimesters for all pregnant women infected with syphilis, regardless of the stage of syphilis [8, 9].

Despite the efficacy of the WHO-recommended treatment regimen (i.e., one course of treatment), little is known about how it compares to China-recommended treatment in clinical practice. Some argue that China's two courses of treatment seem to be redundant in clinical practice. Hong et al. found that the CS incidence was similar (adjusted OR 1.74, 95% CI 0.37-8.26) between pregnant women treated with two courses of BPG and those treated with one course [4]. Similarly, Liu et al. found that syphilis-infected pregnant women treated with one or two courses of penicillin had a similar risk of APOs (adjusted risk ratio 1.36, 95% CI 0.94-1.96) [10]. These two studies have limitations such as investigating only one rare outcome (i.e., CS) or using a small sample size. Conversely, there is evidence suggesting that adequate treatment according to China's recommended regimen is more effective (adjusted OR 0.3, 95% CI 0.1-0.7) in reducing the risk of APOs for syphilis-infected women compared to inadequate treatment (i.e., not receiving standardized treatment in line with the recommendation of China) [11]. The inconsistent evidence makes the practice challenging to adopt a treatment regimen for syphilis-infected pregnant women at the national level. Given the public health priority of syphilis treatment in China, it is necessary to provide empirical evidence on the effectiveness of China-recommended treatment compared to internationally established guidelines. Such evidence will serve to establish a standard treatment regimen for government and clinicians and help to increase treatment compliance among syphilis-infected mothers. Furthermore, limited evidence is available regarding the efficacy of alternative antibiotic medications to penicillin for syphilis-infected pregnant women and the fetus [12, 13].

The developmental origins of health and disease suggest that multiple early factors affect one's health and susceptibility to disease over the life course [14]. Given this context, the large spectrum of APOs beyond the relatively rare outcome of CS should be considered. These include prematurity, LBW, and small for gestational age (SGA). It is also important to investigate other risk factors associated with various APOs in pregnant women with syphilis. These include maternal education, marital status, history of previous infection with syphilis, and syphilis infection status of sexual partner [15]. The effects of these multiple risk factors together with treatment regimens on APOs among pregnant women with syphilis remain unclear.

The primary aim of this present study was to compare the efficacy of different treatment regimens in reducing multiple APOs among pregnant Chinese women with syphilis. We hypothesized that the China-recommended treatment regimen (i.e., two courses) was more efficacious than the WHO-recommended regimen (i.e., one course). A secondary aim was to examine the multiple risk factors associated with multiple APOs in this population.

## 2. Methods

### 2.1. Participants and Study Design

This retrospective cohort study included all syphilis-infected pregnant women registered in China's Information Management System for Prevention of Mother-to-Child Transmission of Syphilis in Guangzhou between July 2011 and June 2018. Pregnant women were screened for antibodies against *Treponema pallidum* using *Treponema pallidum* particle agglutination test (TPPA) at their first antenatal care visit. If this test was positive, the woman was then tested with toluidine red unheated serum test (TRUST). Maternal syphilis was diagnosed by positive TPPA and TRUST. Doctors assessed maternal clinical stages of syphilis (primary, secondary, early latent, or late latent) based on their clinical symptoms and duration of infection and referred them to designated infectious disease and dermatology departments in general hospitals to receive free treatment and testing throughout pregnancy until delivery.

Exclusion criteria included women who (1) were coinfected with HIV, (2) elected to terminate their pregnancy, (3) had a twin or multiple pregnancies, (4) left Guangzhou before delivery, and (5) had incomplete information. Infants born to mothers with syphilis were followed up every 3 months from birth to 18 months, until the diagnosis of CS was excluded or confirmed.

The study was conducted in accordance with China's guidelines. Pregnant women with syphilis infection signed informed consent before being referred to designated institutions for free treatment and regular follow-up. The database downloaded from the national system was anonymous. Only the first and corresponding authors had access to the data. The data analysis and interpretation process excluded pregnant women's personal information (such as phone numbers and identity numbers).

### 2.2. Treatment of Maternal Syphilis

Participants were divided into four groups according to different treatment regimens completed during pregnancy and maternal syphilis clinical stages: (1) no treatment for those who did not receive any antisyphilis treatment; (2) alternatives to penicillin treatment for those who received treatment for syphilis with ceftriaxone or erythromycin; (3) WHO-recommended treatment for those who received BPG in a single dose who were in early stages of syphilis or received 3 doses of BPG who were in the late stages or latent of unknown duration; pregnant women who did not complete the required 3 doses of BPG were excluded, and they were lost to follow-up before delivery; and (4) China-recommended treatment for those who received two courses of BPG treatment.

### 2.3. Adverse Pregnancy Outcomes

APOs included at least one of the following: (1) ectopic pregnancy, defined as fertilized eggs implanted outside the uterine cavity [16]; (2) spontaneous abortion, defined as a spontaneous pregnancy loss at or before 20 weeks of gestation [16]; (3) stillbirth, defined as fetal death occurring after 20 weeks of gestation (or weighing more than 500 grams) and before/during labor (based on a 1-minute Apgar score of 0 [16]); (4) prematurity or LBW, defined as a live birth before a gestational age of 37 weeks or birth weight less than 2500 grams [16]; (5) SGA, defined as a live infant birth weight of less than the 10th percentile by gestational age and sex (based on the INTERGROWTH-21^st^ Project Standards [17]); (6) infant death, defined as the death of a live-born infant within 1 year of age; and (7) CS, defined as having one of the following situations: (i) neonatal nontreponemal antibody titer more than 4-fold the maternal titer before delivery, (ii) reactive treponemal antibody test of IgM, (iii) increased nontreponemal antibody titer and positive treponemal-specific antibody test, and (iv) positive treponemal-specific antibody test after 18 months of age [8].

### 2.4. Data Collection

Standardized questionnaires [8] were used to collect data including sociodemographic, clinical, laboratory, treatment, and obstetric factors of syphilis-infected pregnant women. The clinical and obstetric information of syphilis-positive pregnant women included syphilis stages, duration of infection, history of syphilis infections, route of infection, syphilis status of partners, and coinfection with HBV, HIV, Group B streptococci, or other pathogenic microorganisms. Information on their children, which included birth date, gestational age, sex, birth weight, mode of delivery, birth defects, neonatal diseases, and laboratory tests at ages 3, 6, 9, 12, 15, and 18 months, was collected with assistance from medical professionals. The accuracy of all case cards was evaluated stepwise by district-level and municipal-level staff members who had undergone unified training. Gestational age at birth was expressed as completed weeks and was based on the first- or second-trimester ultrasound. In the absence of a recorded ultrasound result, the last menstrual period was used to calculate gestational age.

### 2.5. Statistical Analysis

Descriptive analyses were used to describe the incidence rate of each APO. To maintain a normal distribution, the titers from maternal TRUST at treatment were transformed using the log_2_ of the reciprocal nontreponemal titer (log_2_ [1/*T*]) [18]. Univariate logistic regression analyses were conducted to identify potential risk factors associated with APOs. Multivariate logistic regression analyses were then conducted to investigate the associations between APOs and potential risk factors. To understand the impact of TRUST titers on pregnancy outcomes, we used the 3^rd^ power simulation smooth line with the highest coefficient of determination (*R*^2^). TRUST results were then divided into high- and low-titer groups, with stratified analysis conducted to further explore the effects of different treatment regimens on APOs. All analyses were conducted using the Statistical Package for the Social Sciences version 20 software package for Windows (SPSS Inc., Chicago, IL, USA). All reported *p* values are based on a two-sided test with a significance level of *α* = 0.05.

## 3. Results

**Figure 1:**
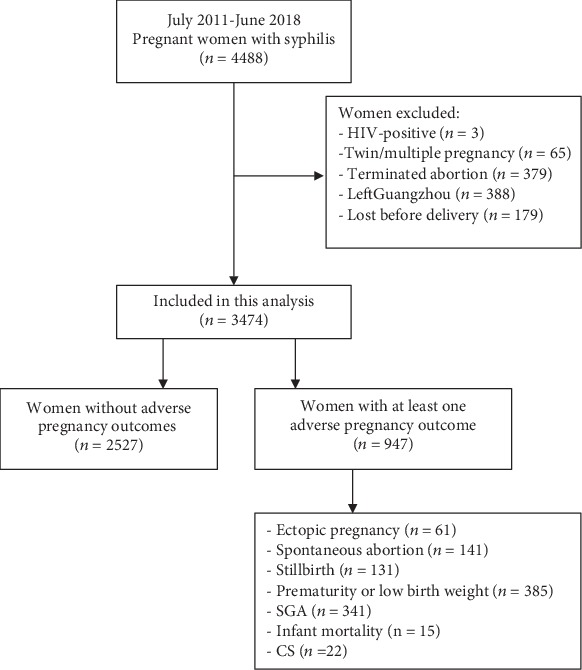
Sample size flow chart for the study analysis. CS: congenital syphilis; SGA: small for gestational age.

We enrolled 3474 out of 4488 pregnant women with syphilis (Figure 1). By the end of the follow-up, more than a quarter of the women (27.3%; *n* = 947) had at least one APO as follows: ectopic pregnancy (1.8%; *n* = 61), spontaneous abortion (4.1%; *n* = 141), stillbirth (3.8%; *n* = 131), prematurity or LBW (11.1%; *n* = 385), SGA (9.8%; *n* = 341), infant death (0.4%; *n* = 15), and CS (0.6%; *n* = 22).

### 3.1. Participants' Demographics and Clinical Characteristics

In the attrition analysis, we found that the study sample had an older age, a higher socioeconomic position, and a higher proportion of no history of APOs (Table 2), than those who were excluded. In our final analysis, 55.3% of the participants were aged 25~34. Around half (54.6%) of the participants received China-recommended treatment, one-quarter (25.8%) received the WHO-recommended treatment, 2.9% received alternatives to penicillin, and 16.7% received no treatment (Table 3).

### 3.2. Univariate Analysis

Pregnant women were more likely to experience APOs if they were younger, were unmarried, had lower educational attainment, had no history of syphilis infections, and had higher levels of log_2_-transformed TRUST titers before treatment (Table 3). Pregnant women experiencing at least one APO were more likely to receive no treatment during pregnancy (27.7%) and less likely to receive the China-recommended treatment regimen (37.6%).

### 3.3. Multivariate Analysis and Stratified Analysis

Multivariate results showed that women who received China-recommended treatment were less likely to have APOs (OR 0.47, 95% CI 0.38-0.57) than those receiving WHO-recommended treatment, whereas the odds of experiencing APOs was elevated by 1.6 times in those with no treatment (95% CI 1.25-2.00). We also found that a high level of log_2_-transformed TRUST titers before treatment was a common risk factor across different APOs (OR 1.14, 95% CI 1.10-1.19) (Table 4).

**Figure 2:**
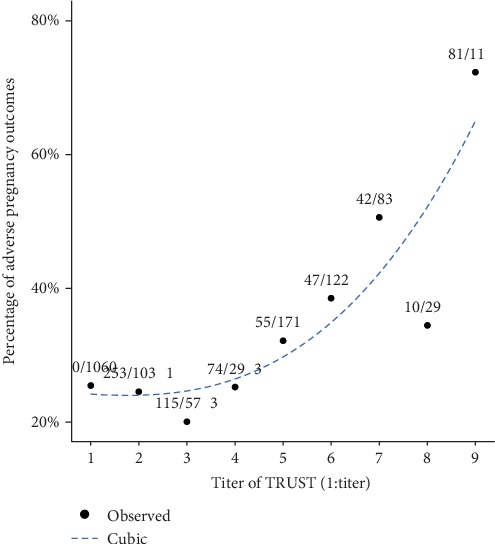
Curve estimation for proportions of adverse pregnancy outcomes in women with syphilis. TRUST: toluidine red unheated serum test.

According to the best fit model (Figure 2), the coefficient of determination (*R*^2^) was 0.9706. The incidence of adverse pregnancy outcomes increased significantly when the titers of TRUST were higher than 1 : 8. Based on this cut-off value, we conducted a stratified analysis and found that the China-recommended treatment was effective in reducing APOs both for those with TRUST ≥ 1 : 8 and for those with TRUST < 1 : 8 (Table 5).

## 4. Discussion

The present study compared the efficacy of different treatment regimens and examined multiple risk factors associated with multiple APOs for Chinese pregnant women with syphilis. Specifically, we found the following: (1) corresponding to our hypothesis, China-recommended treatment might have a larger effect than WHO-recommended treatment in reducing multiple APOs, while alternatives to penicillin treatment had a similar effect to WHO-recommended treatment; (2) a high level of log_2_-transformed TRUST titers before treatment was a common risk factor for multiple APOs, showing that every twofold increase in maternal TRUST titer increased the odds of delivering a newborn who was preterm/LBW or SGA or diagnosed with CS; and (3) syphilis-infected pregnant women who were unmarried, younger than 24 years old, and less educated had higher risks of adverse pregnancy outcomes.

While WHO-recommended treatment has been widely accepted in the international context, we found that the incidence of at least one APO (ectopic pregnancy, spontaneous abortion, stillbirth, and preterm/LBW) among syphilis-infected pregnant women treated with China-recommended treatment was lower than among those treated with WHO-recommended treatment. This indicates that an additional course significantly enhances efficacy. Multiple BPG doses may be associated with more favorable pregnancy outcomes by increasing serum concentrations [13]. The U.S. Preventive Services Task Force and CDC also recommend repeated screening for women at high risk of syphilis, including those living in high-prevalence communities, those living with HIV, those with a history of incarceration or commercial sex work, and those living with an infected partner at 28-32 weeks of gestation and at delivery, and repeated treatment if necessary [12, 19]. Therefore, China's regimen, which recommends repeated treatment with another three doses of BPG, ensures that syphilis-infected pregnant women who require repeated treatment are treated promptly. An alternative explanation could be that the two-course treatment regimen recommended by China can ensure that pregnant women receive treatment as soon as possible (Table 6). However, patients using the one-course treatment recommended by WHO may be treated later than those using two-course treatment recommended by China (Table 6) due to the fact that the WHO regimen lacks a clear time point of treatment initiation [1]. This limitation was addressed by an updated WHO guideline in 2017 [20], emphasizing the importance of initiating treatment in the first trimester. This updated WHO guideline helps to implement the WHO-recommended treatment regimen in the early onset of syphilis infection of pregnant woman in the future. Therefore, the effectiveness of the two-course treatment regimen found in this study may be due to another course of treatment or earlier initiation of treatment. However, the two-course treatment regimen in China may not be applicable to other countries, especially those in shortage of benzathine penicillin.

We did not find that the incidence of CS among women treated with WHO-recommended treatment was significantly different from those treated with China-recommended treatment, consistent with other findings [4, 10, 11]. One possible reason is the low incidence of CS in this study, and further studies are needed to illustrate. Consistent with previous studies [21, 22], we also found that untreated maternal syphilis remained an important risk factor for several APOs. While syphilis-infected pregnant women in China are recommended to have two courses of penicillin for prevention of MTCT, some patients did not receive standardized antisyphilis treatment due to poor reproductive health knowledge or unfamiliarity with the antenatal care system, to low coverage of health insurance, to fear of discrimination by spouse or medical staffs, or to doctors providing one-course treatment [11, 23]. Further research is needed to explore specific reasons why these pregnant women with syphilis infection do not receive treatment and find possible ways to eliminate barriers, with the aim of improving treatment adherence and reducing APOs.

We extended the evidence in the current literature by examining the effectiveness of alternatives to penicillin in reducing APOs. Alternative therapies used for penicillin-allergic individuals are not commonly used during pregnancy because of either transplacental effects (e.g., erythromycin or azithromycin) or adverse fetal effects (e.g., tetracycline and doxycycline) [13]. Ceftriaxone can cross the placental barrier, and the optimal dose and duration of therapy for pregnant women are unknown and may increase the risk of kernicterus in newborns [24]. Although the British guidelines and China's regimen recommend erythromycin, azithromycin, and ceftriaxone as alternative treatments, there is inconsistent evidence that alternative antibiotics are efficacious for the treatment of syphilis during pregnancy [19]. It is widely accepted that penicillin is the optimal drug for the treatment of syphilis. Our findings suggest that alternatives to penicillin treatment have a similar effect on the pregnancy outcomes of syphilis-infected women compared to WHO-recommended treatment, which is consistent with the previous findings of Dou et al. [25]

High titers of nontreponemal antibodies suggest early syphilis, including primary, secondary, and early latent syphilis [26]. We found that every twofold increase in maternal TRUST titer increased the odds for spontaneous abortion and delivering a preterm or LBW, SGA infant, or infant with CS. Two previous studies found a nearly twofold increase in risk for CS with each doubling of nontreponemal titers [26, 27]. A high titer of nontreponemal antibodies (≥1 : 8) represents significant fetal exposure to *Treponema pallidum* [28]. Our study suggests that China-recommended treatment is effective in reducing APOs both for those with TRUST ≥ 1 : 8 and for those with TRUST < 1 : 8, indicating that repeated treatment in the third trimester may be necessary.

The reasons for APOs are likely to be multifactorial. Our study suggests that syphilis-infected women who are unmarried, are younger than 24 years old, and have a low educational level are more likely to experience APOs. Recently, a multifaceted intervention carried out in sub-Saharan Africa suggested that behavior intervention and provision of supplies can lead to more than 95% of women being screened and treated for syphilis [29]. Strengthening health education and health promotion for high-risk groups is a major measure to decrease the incidence of APOs among pregnant women with syphilis infection.

Our study has the following advantages. First, we analyzed multiple adverse pregnancy outcomes in pregnant women with syphilis infections, such as spontaneous abortion, stillbirth, low birth weight/premature, small for gestational age, congenital syphilis, and infant death, as well as multiple factors such as the treatment regimen, TRUST titers before treatment, and maternal demographics. Second, the large sample size is effective to identify a series of adverse pregnancy outcomes and influencing factors. Third, we compare the effect of different treatment regimens on adverse pregnancy outcomes, which is helpful for informing clinicians and patients to implement two courses of treatment. Fourth, our study shows that the cut-off value of TRUST titer before treatment is 1 : 8. This cut-off value is beneficial for work, because it suggests that clinicians should pay attention to the patients with TRUST titer higher than 1 : 8. Various measures should also be conducted to improve their treatment compliance.

This retrospective cohort study has several limitations. First, this study was part of the Project of Prevention of Mother-to-Child Transmission of HIV, Syphilis, and HBV, which is aimed at reducing the incidence of HIV, CS, and HBV. Therefore, the original project was not designed to compare the effects of different treatment regimens, and our classifications regarding treatment groups were based on the treatment they already received and their syphilis stages, rather than a predetermined definition. Therefore, the syphilis-infected pregnant women receiving different treatment regimens were different from each other in terms of demographic and clinical characteristics. For example, those who received different treatment regimens had different marital status which was related to adverse pregnancy outcomes (data is shown in Table 6). These demographic and clinical characteristics might be potential factors influencing adverse pregnancy outcomes regardless of treatment regimens, and we analyzed the impact of these factors on adverse pregnancy outcomes. Second, data were obtained from surveillance registry, with some information missing or incomplete, and patients excluded from this study were in a lower social position than those included in this study (data is shown in Table 2), which needs caution when making conclusions. Third, the small numbers of CS and neonatal death might have limited the power of the study to detect differences in risk factors that have been identified in previous studies (e.g., current syphilis status of women's partners). Fourth, we did not collect other important information (e.g., income, health insurance, risk behaviors, and nutritional status during pregnancy) of the pregnant women, which may also contribute to adverse pregnancy outcomes.

This study has several policy and research implications. At the policy level, the government should strengthen the management of key populations (such as unmarried, younger, and low education) to improve their compliance with treatment. It may be more efficacious for pregnant women with syphilis to receive the two-course treatment recommended by China, but it is also very important for areas where penicillin is scarce to adopt the treatment regimen recommended by WHO as soon as possible [20]. In further informing actionable policy opportunities to reduce APOs among pregnant women with syphilis infection, the breadth of existing longitudinal and multilevel cohort data can enable the generation of policy-relevant findings quickly and cost-effectively [30]. Future work can apply a novel statistical approach such as the causal mediation analysis based on the interventional effect [31] to answer the effect of the China-recommended treatment regimen on APOs via potential mediators such as patients' health literacy and treatment compliance among pregnant women with syphilis infection.

## 5. Conclusions

Syphilis remains a major contributor to newborn morbidity, mortality, and adverse obstetrical outcomes, especially in the context of pregnant women with high titers of nontreponemal antibodies. Pregnancy outcomes of syphilis-infected pregnant women can be improved by receiving the standardized treatment regimen recommended by the Chinese government. This highlights the need for national implementation of the Chinese guidelines.

## Figures and Tables

**Table 1:** Different treatment regimens for pregnant women with syphilis prescribed by WHO, USA, UK, and China.

Country, year	Therapy regimens	Primary, secondary, or early latent < 1 year	Latent > 1 year, latent of unknown duration
WHO, 2003 [1]; USA, 2015 [6]	Recommended regimens	Benzathine penicillin G 2.4 million units intramuscular (IM) in a single dose	Benzathine penicillin G 7.2 million units total, administered as 3 doses of 2.4 million units IM each at 1-week intervals
	If with penicillin allergy	Pregnant women who are allergic to penicillin should be desensitized and treated with penicillin	

UK, 2015 [7]	Recommended regimens	Benzathine penicillin G 2.4 million units IM single dose in the first and second trimesters. When maternal treatment is initiated in the third trimester, a second dose of benzathine penicillin G 2.4 million units IM should be given after one week (day 8)	Benzathine penicillin G 2.4 million units IM weekly on days 1, 8, and 15 (three doses)
	Alternative treatments	(1) Procaine penicillin G 600,000 units IM. Daily for 10 days (2) Amoxicillin 500 milligrams (mg) orally four times a day (q.d.s.) plus probenecid 500 mg orally q.d.s. for 14 days (3) Ceftriaxone 500 mg IM daily for 10 days (4) Erythromycin 500 mg orally q.d.s. for 14 days (5) Azithromycin 500 mg orally daily for 10 days	(1) Procaine penicillin G 600,000 units IM once a day for 14 days (2) Amoxicillin 2 grams (g) orally thrice a day (t.d.s.) plus probenecid 500 mg q.d.s. for 28 days

China,2011 [9]; China, 2015 [8]	Recommended regimens	(1) Benzathine penicillin G 2.4 million units IM weekly, 3 doses for 1 course (2) Procaine penicillin G 0.8 million units IM daily, 15-20 days for 1 course Standardized treatment should meet the three conditions at the same time: (a) One of the above regimens was used (b) Two courses of treatment were given during pregnancy, and the interval between the two courses was more than 2 weeks (3) The second course was performed and completed in the third trimester	
	Alternative treatments	(1) Ceftriaxone, 1 g daily, intramuscular or intravenous, 10-14 days for 1 course (2) Erythromycin 500 mg, 4 times daily, orally, 15-30 days for 1 course Two courses of treatment given during pregnancy, with the interval between the two courses more than 2 weeks If pregnant women were given the above alternative treatments, they were considered to have received nonstandardized treatment	

**Table 2:** Comparison of demographic and clinical characteristics between pregnant women with syphilis who were included and excluded in this study (*n*, %).

Characteristics	Included subjects (*n* = 3474)	Excluded subjects (*n* = 1014)	*t*/*χ*^2^ value	*p* value
Mother's age in years (mean ± SD)	30.65 ± 6.01	29.65 ± 6.40	-4.59	<0.001
Marital status				
Married	3018 (86.9)	801 (79.0)	38.42	<0.001
Unmarried	456 (13.1)	213 (21.0)		
Ethnicity				
Han	3250 (93.6)	917 (90.4)	11.49	0.001
Others	224 (6.5)	97 (9.6)		
Educational attainment				
Bachelor or above	413 (13.2)	78 (7.7)	57.06	<0.001
High school	872 (27.8)	272 (26.8)		
Middle school	1484 (47.3)	463 (45.7)		
Primary school or below	367 (11.7)	201 (19.8)		
Employed				
Yes	973 (34.8)	293 (28.9)	11.65	0.001
No	1824 (65.2)	721 (71.1)		
Migrant resident				
No	1084 (31.2)	182 (18.0)	68.09	<0.001
Yes	2390 (68.8)	832 (82.1)		
Primipara				
Yes	1230 (35.4)	508 (50.1)	71.41	<0.001
No	2244 (64.6)	506 (49.9)		
History of APOs^∗^				
No	3202 (92.2)	880 (86.8)	27.67	<0.001
Yes	272 (7.8)	134 (13.2)		
History of syphilis infections				
No	2017 (58.1)	672 (66.3)	22.04	<0.001
Yes	1457 (41.9)	342 (33.7)		
Maternal syphilis stage				
Latent	2729 (78.6)	743 (73.3)	14.89	0.001
Stages I-III	184 (5.3)	55 (5.4)		
Unknown	561 (16.2)	216 (21.3)		
Current syphilis status (partner)				
Negative	855 (24.6)	192 (28.1)	7.21	0.027
Positive	241 (6.9)	59 (8.6)		
Unknown	2378 (68.5)	433 (63.3)		
Log_2_ titers of TRUST before treatment (mean ± SD)	1.74 ± 2.03	1.67 ± 1.70	-0.92	0.358

^∗^APOs included spontaneous abortion, stillbirth, and preterm birth. SD: standard deviation; TRUST: toluidine red unheated serum test.

**Table 3:** Demographic and clinical characteristics of pregnant women with syphilis (*n*, %).

Characteristic	Total sample (*n* = 3474)	Adverse pregnancy outcomes			
		No (*n* = 2527)	Yes (*n* = 947)	*t*/*χ*^2^ value	*p* value
Mother's age in years				8.885	0.012
≤24	586 (16.9)	398 (15.7)	188 (19.9)		
25~34	1922 (55.3)	1408 (55.7)	514 (54.3)		
≥35	966 (27.8)	721 (28.5)	245 (25.9)		
Marital status				23.207	<0.001
Married	3018 (86.9)	2238 (88.6)	780 (82.4)		
Unmarried	456 (13.1)	289 (11.4)	167 (17.6)		
Ethnicity				1.923	0.166
Han	3250 (93.6)	2373 (93.9)	877 (92.6)		
Others	224 (6.5)	154 (6.1)	70 (7.4)		
Educational attainment				11.701	0.008
Bachelor or above	413 (13.2)	321 (14.0)	92 (10.9)		
High school	872 (27.8)	632 (27.6)	240 (28.4)		
Middle school	1484 (47.3)	1092 (47.7)	392 (46.4)		
Primary school or below	367 (11.7)	246 (10.7)	121 (14.3)		
Employed				1.148	0.701
Yes	973 (34.8)	712 (34.6)	261 (35.4)		
No	1824 (65.2)	1347 (65.4)	477 (64.6)		
Migrant resident				0.285	0.593
No	1084 (31.2)	795 (31.5)	289 (30.5)		
Yes	2390 (68.8)	1732 (68.5)	658 (69.5)		
Primipara				2.722	0.099
Yes	1230 (35.4)	874 (34.6)	356 (37.6)		
No	2244 (64.6)	1653 (65.4)	591 (62.4)		
History of APOs				0.689	0.406
No	3202 (92.2)	2335 (92.4)	867 (91.6)		
Yes	272 (7.8)	192 (7.6)	80 (8.5)		
History of syphilis infections				6.561	0.010
No	2017 (58.1)	1434 (56.8)	583 (61.6)		
Yes	1457 (41.9)	1093 (43.3)	364 (38.4)		
Maternal syphilis stage				0.413	0.814
Latent	2729 (78.6)	1992 (78.8)	737 (77.8)		
Stages I-III	184 (5.3)	132 (5.2)	52 (5.5)		
Unknown	561 (16.2)	403 (16.0)	158 (16.7)		
Partner's current syphilis status				19.598	<0.001
Negative	855 (24.6)	670 (26.5)	185 (19.5)		
Positive	241 (6.9)	179 (7.1)	62 (6.6)		
Unknown	2378 (68.5)	1678 (66.4)	700 (73.9)		
Gestational week at syphilis diagnosis (mean ± SD)	18.65 ± 9.86	18.80 ± 9.85	18.25 ± 9.87	1.420	0.156
Gestational week at treatment initiation (mean ± SD)	20.34 ± 10.13	20.28 ± 10.05	20.54 ± 10.36	0.497	0.560
Syphilis treatment during pregnancy				183.967	<0.001
WHO-recommended treatment	897 (25.8)	592 (23.4)	305 (32.2)		
China-recommended treatment	1897 (54.6)	1541 (61.0)	356 (37.6)		
Alternatives to penicillin treatment	100 (2.9)	76 (3.0)	24 (2.5)		
No treatment	580 (16.7)	318 (12.6)	262 (27.7)		
Log_2_ titers of TRUST before treatment (mean ± SD)	1.74 ± 2.03	1.53 ± 1.73	2.29 ± 2.59	8.453	<0.001
Mode of delivery				50.051	<0.001
Spontaneous labor	1834 (58.4)	1461 (57.8)	373 (60.8)		
Elective Caesarean section	755 (24.0)	665 (26.3)	90 (14.7)		
Emergency Caesarean section	552 (17.6)	401 (15.9)	151 (24.6)		
Gestational age at delivery in weeks (mean ± SD)	39.12 ± 1.98	39.55 ± 1.19	37.35 ± 3.23	16.618	<0.001
Birth weight in grams (mean ± SD)	3146.43 ± 497.40	3294.04 ± 371.66	2538.92 ± 488.47	35.866	<0.001
Newborn's sex				4.724	0.030
Girl	1427 (45.4)	1124 (44.5)	303 (49.4)		
Boy	1714 (54.6)	1403 (55.5)	311 (50.7)		

^∗^APOs included spontaneous abortion, stillbirth, and preterm birth. CI: confidence interval; SD: standard deviation; APOs: adverse pregnancy outcomes; TRUST: toluidine red unheated serum test.

**Table 4:** Risk factors associated with different adverse pregnancy outcomes in women with syphilis (multivariate analysis) (odds ratio and its 95% confidence interval).

Characteristic	At least one APO^∗^	Ectopic pregnancy^∗^	Spontaneous abortion^#^	Stillbirth	Preterm/LBW	SGA	CS
Maternal age	0.99 (0.98-1.00)	1.08 (1.03-1.14)	1.01 (0.98-1.05)	0.96 (0.93-0.99)	1.00 (0.98-1.02)	0.98 (0.96-1.00)	0.93 (0.85-1.01)
Marital status (ref = married)	1.32 (1.03-1.69)	4.90 (2.50-9.61)	1.87 (1.14-3.07)	0.85 (0.48-1.48)	1.22 (0.86-1.72)	0.81 (0.56-1.19)	0.96 (0.27-3.33)
Educational attainment (ref = bachelor or above)							
High school	1.16 (0.87-1.55)	1.09 (0.37-3.23)	1.86 (0.92-3.73)	1.44 (0.72-2.86)	1.02 (0.68-1.53)	0.93 (0.61-1.42)	0.35 (0.09-1.30)
Middle school	1.06 (0.81-1.39)	0.85 (0.30-2.38)	1.06 (0.53-2.10)	1.04 (0.53-2.03)	0.99 (0.68-1.44)	1.16 (0.79-1.70)	0.23 (0.06-0.83)
Primary school or below	1.25 (0.89-1.76)	1.51 (0.50-4.54)	0.56 (0.23-1.35)	1.34 (0.61-2.96)	1.17 (0.73-1.87)	1.42 (0.88-2.31)	0.58 (0.14-2.41)
History of syphilis infections (ref = no)	1.12 (0.94-1.34)	1.13 (0.61-2.09)	1.18 (0.78-1.77)	0.95 (0.63-1.43)	1.29 (1.01-1.66)	0.84 (0.65-1.09)	2.32 (0.89-6.03)
Partner's current syphilis status (ref = negative)							
Positive	1.08 (0.76-1.54)	1.54 (0.44-5.41)	0.87 (0.37-2.04)	0.65 (0.24-1.74)	1.20 (0.72-2.01)	0.99 (0.61-1.63)	0.66 (0.06-7.22)
Unknown	1.22 (0.99-1.49)	1.04 (0.46-2.32)	0.90 (0.56-1.46)	1.15 (0.72-1.83)	1.51 (1.12-2.05)	0.92 (0.70-1.22)	1.84 (0.50-6.82)
Syphilis treatment during pregnancy (ref = WHO-recommended treatment)							
China-recommended treatment	0.47 (0.38-0.57)	0.03 (0.04-0.21)	0.11 (0.05-0.21)	0.40 (0.26-0.63)	0.52 (0.40-0.69)	0.91 (0.69-1.21)	0.49 (0.15-1.62)
Alternatives to penicillin treatment	0.63 (0.37-1.06)	—	—	0.43 (0.10-1.81)	0.84 (0.43-1.62)	1.16 (0.59-2.30)	1.77 (0.20-16.06)
No treatment	1.58 (1.25-2.00)	3.59 (1.96-6.57)	3.46 (2.30-5.20)	1.18 (0.74-1.90)	0.97 (0.69-1.38)	0.84 (0.55-1.27)	1.59 (0.52-4.88)
Log_2_ titers of TRUST before treatment	1.14 (1.10-1.19)	0.89 (0.78-1.02)	1.08 (1.00-1.16)	1.05 (0.97-1.14)	1.19 (1.13-1.25)	1.10 (1.04-1.16)	1.34 (1.16-1.58)
Mode of delivery (ref = spontaneous labor)							
Elective Caesarean section	—	—	—	—	0.62 (0.44-0.87)	0.65 (0.47-0.91)	2.34 (0.72-7.58)
Emergency Caesarean section	—	—	—	—	1.82 (1.38-2.40)	1.29 (0.95-1.74)	1.21 (0.38-3.83)
Gestational age at delivery in weeks	—	—	—	—	—	—	0.97 (0.78-1.20)
Birth weight in grams	—	—	—	—	—	—	1.00 (1.00-1.00)
Newborn's sex (ref = girl)	—	—	—	—	0.85 (0.67-1.07)	—	0.90 (0.36-2.28)

APO: adverse pregnancy outcome; LBW: low birth weight; SGA: small for gestational age; CS: congenital syphilis; TRUST: toluidine red unheated serum test. ^∗^The sample size for alternatives to penicillin treatment was one. ^#^The sample size for alternatives to penicillin treatment was zero.

**Table 5:** Odds of APOs among syphilis-seropositive pregnant women with high and low titers of TRUST.

Treatment regimen	At least one APO, *n* (%)		Ectopic pregnancy		Spontaneous abortion		Stillbirth		Preterm/LBW	
	*n* (%)	Adjusted OR (95% CI)^#^	*n* (%)	Adjusted OR (95% CI)^#^	*n* (%)	Adjusted OR (95% CI)^#^	*n* (%)	Adjusted OR (95% CI)^#^	*n* (%)	Adjusted OR (95% CI)^#^
TRUST < 1 : 8 (*N* = 2664)										
WHO-recommended treatment	178 (27.1)	Reference	14 (2.1)	Reference	31 (4.7)	Reference	28 (4.3)	Reference	66 (11.3)	Reference
China-recommended treatment	285 (18.8)	0.61 (0.49-0.77)	0	—	10 (0.7)	0.13 (0.06, 0.26)	40 (2.6)	0.56 (0.33, 0.96)	127 (8.7)	0.78 (0.55, 1.11)
Alternatives to penicillin treatment	16 (20.5)	0.73 (0.39-1.37)	1 (1.3)	—	0	—	1 (1.3)	0.37 (0.05, 2.79)	9 (11.8)	1.16 (0.52, 2.58)
No treatment	159 (38.3)	1.82 (1.37-2.41)	30 (7.2)	3.91 (1.93, 7.94)	49 (11.8)	2.83 (1.72, 4.67)	20 (4.8)	1.29 (0.70, 2.39)	46 (14.6)	1.38 (0.89, 2.15)
TRUST ≥ 1 : 8 (*N* = 810)										
WHO-recommended treatment	127 (52.9)	Reference	8 (3.3)	Reference	12 (5.0)	Reference	21 (8.8)	Reference	66 (33.2)	Reference
China-recommended treatment	71 (18.5)	0.21 (0.14-0.31)	0	—	0	—	5 (1.3)	0.15 (0.06, 0.43)	36 (9.5)	0.22 (0.14, 0.36)
Alternatives to penicillin treatment	8 (36.4)	0.48 (0.18-1.27)	0	—	0	—	1 (4.5)	0.61 (0.08, 4.92)	5 (23.8)	0.58 (0.18, 1.87)
No treatment	103 (62.4)	1.37 (0.89-2.12)	8 (4.8)	2.21 (0.70, 6.92)	39 (23.6)	6.36 (3.01, 13.47)	15 (9.1)	1.07 (0.51, 2.24)	30 (29.1)	0.72 (0.41, 1.28)

^#^Adjusted for age, marital status, education, history of syphilis infection, and current syphilis status of partner. APO: adverse pregnancy outcome; TRUST: toluidine red unheated serum test; OR: odds ratio; CI: confidence interval.

**Table 6:** Comparison of demographic and clinical characteristics among syphilis-infected pregnant women receiving different treatment regimens (*n*, %).

Characteristics	WHO-recommended treatment (*n* = 897)	China-recommended treatment (*n* = 1897)	Alternatives to penicillin treatment (*n* = 100)	No treatment (*n* = 580)	*F*/*χ*^2^ value	*p* value
Mother's age in years (mean ± SD)	30.68 ± 6.30	30.65 ± 5.83	30.20 ± 6.58	30.68 ± 6.04	0.199	0.897
Marital status						
Married	770 (85.8)	1695 (89.4)	77 (77.0)	476 (82.1)	31.344	<0.001
Unmarried	127 (14.2)	202 (10.6)	23 (23.0)	104 (17.9)		
Ethnicity						
Han	820 (91.4)	1790 (94.4)	98 (98.0)	542 (93.4)	12.127	0.007
Others	77 (8.6)	107 (5.6)	2 (2.0)	38 (6.6)		
Educational attainment						
Bachelor or above	63 (7.9)	288 (16.4)	9 (10.6)	53 (10.6)	83.644	<0.001
High school	224 (28.1)	496 (28.3)	27 (31.8)	125 (25.0)		
Middle school	378 (47.4)	829 (47.3)	37 (43.5)	240 (47.9)		
Primary school or below	132 (16.6)	140 (8.0)	12 (14.1)	83 (16.6)		
Employed						
Yes	227 (32.6)	571 (36.2)	23 (31.5)	152 (33.7)	3.541	0.315
No	470 (67.4)	1005 (63.8)	50 (68.5)	299 (66.3)		
Migrant resident						
No	214 (23.9)	686 (36.2)	27 (27.0)	157 (27.1)	49.722	<0.001
Yes	683 (76.1)	1211 (63.8)	73 (73.0)	423 (72.9)		
Primipara						
Yes	259 (28.9)	773 (40.7)	41 (41.0)	157 (27.1)	59.405	<0.001
No	638 (71.1)	1124 (59.3)	59 (59.0)	423 (72.9)		
History of APOs^∗^						
No	830 (92.5)	1740 (91.7)	96 (96.0)	536 (92.4)	2.766	0.429
Yes	67 (7.5)	157 (8.3)	4 (4.0)	44 (7.6)		
History of syphilis infections						
No	596 (66.4)	984 (51.9)	54 (54.0)	383 (66.0)	71.552	<0.001
Yes	301 (33.6)	913 (48.1)	46 (46.0)	197 (34.0)		
Maternal syphilis stage						
Latent	671 (74.8)	1583 (83.4)	73 (73.0)	402 (69.3)	81.943	<0.001
Stages I-III	48 (5.4)	99 (5.2)	7 (7.0)	30 (5.2)		
Unknown	178 (19.8)	215 (11.3)	20 (20.0)	148 (25.5)		
Current syphilis status (partner)						
Negative	180 (20.1)	569 (30.0)	26 (26.0)	80 (13.8)	89.710	<0.001
Positive	57 (6.4)	146 (7.7)	8 (8.0)	30 (5.2)		
Unknown	660 (73.6)	1182 (62.3)	66 (66.0)	470 (81.0)		
Gestational week at syphilis diagnosis (mean ± SD)	23.42 ± 11.44	15.72 ± 6.97	21.21 ± 11.16	20.72 ± 11.74	153.194	<0.001
Gestational week at treatment initiation (mean ± SD)	26.48 ± 11.10	16.46 ± 6.58	22.45 ± 10.82	—	417.511	<0.001
Log_2_ titers of TRUST before treatment (mean ± SD)	1.87 ± 2.09	1.50 ± 1.67	1.69 ± 2.04	2.32 ± 2.74	26.683	<0.001

^∗^APOs included spontaneous abortion, stillbirth, and preterm birth. SD: standard deviation; TRUST: toluidine red unheated serum test.

## Data Availability

No additional data are available.
